# Amyotrophic Lateral Sclerosis and Primary Biliary Cirrhosis Overlap Syndrome: Two Cases Report

**DOI:** 10.3389/fneur.2019.00890

**Published:** 2019-08-14

**Authors:** Haibo Zhang, Yaling Liu, Zhenfei Li, Na Liang, Xiaomeng Zhou, Xiangyu Nie, Ting Zhang, Weijing Qi

**Affiliations:** ^1^Department of Neurology, The Second Hospital of Hebei Medical University, Shijiazhuang, China; ^2^Key Laboratory of Neurology of Hebei Province, Shijiazhuang, China; ^3^Department of Neurology, Baoding First Central Hospital of Hebei Province, Baoding, China

**Keywords:** amyotrophic lateral sclerosis, primary biliary cirrhosis, immunological mechanism, mitochondrial mechanism, ursodeoxycholic acid

## Abstract

Amyotrophic lateral sclerosis (ALS) is a disease of which the underlying etiology and pathogenesis are unknown. Numerous data indicate an important role of the immune system and mitochondrial function in the disease. Primary biliary cirrhosis (PBC) is an autoimmune liver disease resulting from a combination of genetic and environmental risk factors. Patients with PBC develop innate and adaptive immune reactions against mitochondrial antigens. Therefore, common mechanisms could exist in both diseases. We present two cases of ALS with PBC to explore the relationship between the two diseases from the immunological and mitochondrial aspects. Further attention should be given to immune-modulating therapy in ALS patients.

## Introduction

Primary biliary cirrhosis (PBC) is an autoimmune cholestatic disease characterized by the non-suppurative destruction of intrahepatic small bile ducts which can eventually progress to liver cirrhosis (1). It mainly affects middle-aged females, and the female to male ratio is about 10:1 (1). A diagnostic serum marker for PBC is an anti-mitochondrial antibody (AMA) which is positive in more than 90% of the patients (2). Autoimmunity plays an important role in the pathogenesis of PBC (3, 4).

Amyotrophic lateral sclerosis (ALS) is a progressive neurodegenerative disorder involved with the upper (brain, brainstem, and spinal cord) and lower (cranial nerve nuclei, anterior horn cells of spinal cord) motor neurons (5). It is characterized clinically by progressive muscle atrophy, muscle weakness, and respiratory insufficiency with a fatal course (6, 7). The median survival duration is 3–5 years after the onset of the disease, while 10% of the patients can survive for over 10 years (8). The proposed hypotheses for the pathogenesis include glutamate excitotoxicity (9), mitochondrial dysfunction (10), gene defects (11), free radical-mediated oxidative stress (12) and immunological mechanism (13). To date, many patients with ALS and immune diseases (multiple sclerosis, myasthenia gravis, etc) have been reported (14), but ALS concomitant with PBC hasn't been reported.

Here we firstly report two cases of ALS with PBC and analyze the possible relationship between them, mainly from immunological, and mitochondrial aspects.

## Case Reports

### Case 1

A 47-year-old female was admitted to the office with limbs weakness and dysarthria in February 2017. She firstly presented with left upper limb weakness in October 2016. Her symptoms deteriorated rapidly, and 2 months later, she suffered from mild dysarthria and sometimes choking while drinking water, difficulty in lifting and fastening buttons, and walking <100 meters. She reported no weight loss during the last 4 months. She had no remarkable past medical history.

During hospitalization, her vitals were stable. On neurological physical examination, the patient had no obvious muscular atrophy but had fasciculations noted in bilateral bicep and tricep muscles. Power was Medical Research Council (MRC) grade 3/5 in the bilateral upper extremities and 4/5 in the lower extremities. Tendon reflexes were 4+ in all extremities. She had hyperpharyngeal reflex and palmomental reflex. Bilateral Hoffman signs were positive. Neither sensory nor cerebellar dysfunction were identified. A complete blood count, serum biochemical studies, thyroid function, tumor marker showed normal results. Hepatitis panel was negative. Antinuclear antibody (ANA) was positive at a titer of 1:3,200 and AMA was over 1:40. The laboratory test showed that levels of immunoglobulins were within normal limits and alexins were almost within normal range. To rule out Sjogren's syndrome, we ordered Saliva Flow Rate (SFR), corneal fluorescein staining (CFS), breaking up time (BUT), Schirmer I test (SIT), anti-Sjogren syndrome A (SSA) antibody, and anti-Sjogren syndrome B (SSB) antibody. The results were all negative. The magnetic resonance imaging (MRI) of the brain and cervical spinal cord showed no abnormalities. Her chest computed tomography (CT) showed multiple subpleural inflammatory nodules. Considering the absence of cough and fever, we advised her to have a regular examination. The upper abdominal CT was suggestive of splenomegaly and liver cirrhosis. It showed that the morphology of the liver was abnormal, the velamen was not smooth, and the surface was rough. Multiple enlarged lymph nodes were observed near porta hepatis. No expansion or stenosis was observed in the intrahepatic and extrahepatic bile ducts. Electromyography (EMG) showed active and chronic denervation in all limbs, and in the sternocleidomastoid and paraspinal muscles. Nerve conduction studies (NCS) revealed decreased compound muscle action potential (CMAP) amplitudes of right median and ulnar nerve. Ursodeoxycholic acid (UDCA), a hydrophilic tertiary bile acid as the first line treatment of PBC, and riluzole were prescribed. In the late follow-up by telephone, she showed bed-bound at home, dysphagia, and weight loss of 40 kg a year after the symptom's onset.

### Case 2

The second case was a 64-year-old woman diagnosed with PBC in June 2010, when she started UDCA 750 mg/d. Some months later, she was started on endoscopic sclerotherapy and injection of cyanoacrylate glue for gastric fundal varices. In December 2017, gastroesophageal varicose vein ligation and stripping were demonstrated for uncontrolled gastric bleeding. She had a 3-year history of type 2 diabetes mellitus, treated by keeping an appropriate diet for blood sugar. She developed dysphonia and weakness of the hands 3 months before admission. The symptoms gradually progressed. Two months later, she presented weakness in her lower limbs, therefore, she was admitted to our hospital in May 2018. She had lost 10 kg of weight over 3 months. She had no family history of neurodegenerative diseases.

**Figure 1:**
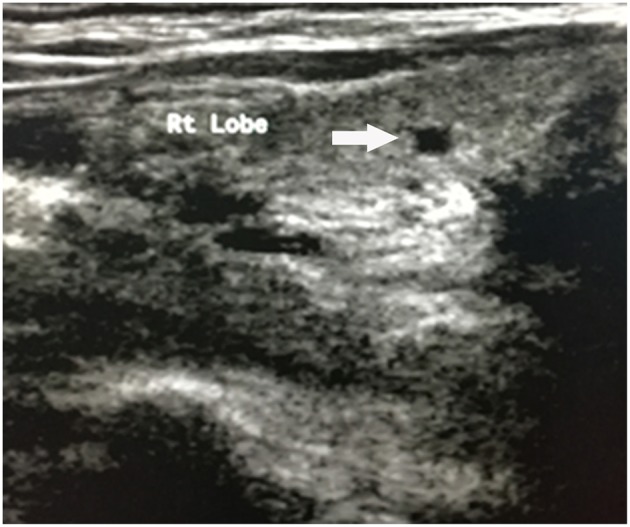
Hypoechoic nodule of right lobe of thyroid (white arrow).

**Figure 2:**
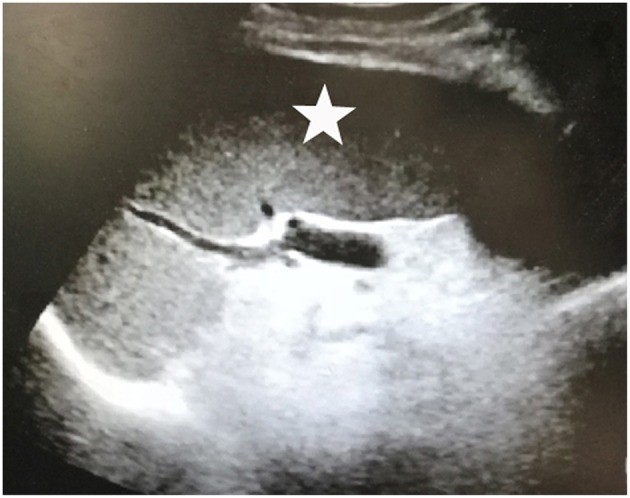
Splenic enlargement (white asterisk).

Upon physical examination, muscle atrophy was observed bilaterally in the first dorsal interosseous muscles and the thenar, hypothenar muscles. Fasciculations and atrophy were evident in the tongue. Power was MRC grade 3/5 in the upper limbs and 4/5 in the lower limbs bilaterally. Deep tendon reflexes were brisk in all extremities. Positive bilateral Hoffman signs and hyperreflexia in the pharyngeal muscles were observed. No abnormality was observed in sensations and cerebellar function. AMA (>1:40) and ANA (1:320) were both positive. Hepatitis markers were negative, so were tumor markers. The levels of white blood cell count (1.80^*^109/L), red blood cell count (3.11^*^1012/L), hemoglobin (105 g/L), and platelet (30^*^109/L) all decreased. The biochemical results showed aspartate aminotransferase (AST) 41.3U/L and total bilirubin (TBIL) 26.0 umol/L. Baseline data of the patient was as follows (Table 1). There were some lacunar infarctions in bilateral frontal, temporal lobes, and left basilar ganglia on brain MRI. A thyroid ultrasound scan showed small nodules on the left and right lobes (Figure 1). Clear lungs were observed on her chest CT. Abdominal ultrasound visualized out of proportion hepatic lobes, mild heterogeneous decrease in echogenicity of the portal vein consistent with mural thrombus, splenomegaly (Figure 2) and dilated splenic vein, neither biliary obstruction nor space-occupied lesions. The CMAP amplitudes of right median and ulnar nerve decreased on NCS. EMG revealed florid active denervation changes in bulbar muscles and all limbs. Lumbar puncture was not executed because of her low platelet count. She was given UDCA, riluzole, and edaravone (Radicava), a new medication for ALS in 2017 approved by Food and Drug Administration (FDA) (15). At the follow-up, she had indwelled gastric tube and difficulty in ambulation in 8 months.

**Table 1:** Baseline data of the patient in case 2.

**Laboratory values**	
**Blood routine**	**Hepatitis screening**
White blood cell count, 1.80^*^10^9^/L (3.50–9.50)	HBsAg ELISA Negative
Red blood cell count, 3.11^*^10^12^/L(3.80–5.10)	Anti-HCV ELISA Negative
Hemoglobin, 105 g/L (115–150)	Anti-HBc Total Negative
Platelet, 30^*^10^9^/L(125–350)	HIV ELISA Negative
**Coagulation routine**	**Immune indices**
International normalized ratio, 1.02 (0.80–1.20)	Immunoglobulin G, 15.5 g/L (7–16)
Activated partial thromboplastin time, 28.9s (25.4–38.4)	Immunoglobulin A, 2.21 g/L (0.7–4.0)
Thrombin time, 13.1 s (10.3–16.6)	Immunoglobulin M, 1.22 g/L (0.4–2.3)
**Biochemistry**	Antinuclear antibody, Positive (1:320)
Aspartate aminotransferase, 41.3 U/L (13–35)	Anti-mitochondrial antibody, Positive (>1:40)
Alanine aminotransferase, 31.4 U/L (7–40)	Alexin C3, 0.74 g/L (0.80–1.60 g/L)
r-Glutamyltransferase, 101 U/L (7–45)	Alexin C4, 0.18 g/L (0.16–0.38 g/L)
Alkaline phosphatase, 112 U/L (50–135)	**Thyroid function**
Albumin, 39.7 g/L (40–55)	Free triiodothyronine, 4.15 pmol/L (2.76–6.45)
Total bilirubin, 26.0 umol/L (3.4–17.1)	Free thyroxine, 12.80 pmol/L (8.75–22.00)
Creatinine, 48.1 umol/L (41–81)	Thyroid-stimulating hormone, 2.92 mIU/L (0.35–4.31)
**Tumor marker**	**Other indicators**
Alpha fetoprotein, <0.61 ng/ml(0–10.9)	Blood ammonia, 35 umol/L(9–30)
Carcino-embryonic antigen, 2.05 ng/ml(0–10)	Glycated hemoglobin, 8.00% (4.00–6.00)
Glycogen antigen CA125, 34.78 U/ml(0–35)	Folic acid, 3.43 ng/ml (>3.38)
Glycogen antigen CA199, 35.3 U/ml (0–37)	VitaminB12, 640 pg/ml (211–911)

## Discussion

Our patients both presented dysarthria and limbs weakness. One was a limb onset, while the other was bulbar. The disease courses were short. On physical examination, pharyngeal reflex, and tendon reflexes were active or hyperactive. EMG showed denervation changes in all 4 body segments including bulbar, cervical, lumbar, and paraspinal. The clinically-definite diagnosis of ALS requires the presence of combined upper (UMN) and lower motor neurons (LMN) signa, and/or symptoms in at least 3 body segments (16). Therefore, our patients were consistent with clinically-definite ALS.

The diagnosis of PBC requires the exclusion of other liver diseases, no evidence of extrahepatic biliary tract obstruction on imaging, and two of the three criteria are met:(i) AMA titer higher than 1:40, (ii) alkaline phosphatase (ALP) over 1.5 times normal upper limit for 24 weeks at least, (iii) characteristic liver histology, especially non-suppurative cholangitis and interlobular bile duct injury (17, 18). Our first patient was a middle-aged female diagnosed with PBC during hospitalization. Considering her negative results of hepatotropic virus, we ruled out cirrhosis caused by long-term hepatotropic virus infection. And no obstruction was found in extrahepatic bile duct on abdominal CT. AMA was positive in 95% of patients with PBC and ANA was 70%. In our case, AMA was positive (> 1:40) and ANA tire reached up to 1:3,200. The results of SFR, CFS, BUT, SIT, rheumatism factor, and lupus anticoagulant were all negative which excluded Sjogren's syndrome, systemic lupus erythematosus, and rheumatoid arthritis. The patient refused a liver biopsy. It had been found that up to 0.5% of the population in screening studies were AMA positive, typically, 50% of those having normal liver biochemistry (19). Our patient was initially diagnosed as PBC though her liver enzymes were normal. The second case had a history of PBC, and started a long-term use of UDCA. She presented end-stage liver performance, like liver cirrhosis, portal hypertension, hypersplenism, bleeding disorder, esophageal-gastric varices, and abnormal blood ammonia risk of hepatic encephalopathy (20).

It had been reported that 70% of PBC patients had extrahepatic diseases, but none with ALS. One study showed that PBC patients with overlapping characteristics of autoimmune hepatitis (AIH) would progress rapidly to cirrhosis and liver failure (21). Whereas, another study suggested that patients with superimposed features were more prone to develop esophageal varices, ascites and liver failure compared with typical PBC patients (22). Therefore, we analyzed the disease characteristics of ALS overlapped with PBC: (i) both patients were female, possibly due to the significant female susceptibility to PBC (1), and some scholars suggested that the gender difference might be related to the presence of genes to control immune tolerance on X chromosome (23) and sex hormone levels (24), (ii) both had a short course of disease, 4 and 3 months, respectively, (iii) clinical signs were involved with upper and LMN, and EMG showed wide denervation, (iv) in view of the follow-up, they both progressed quickly. The second patient might progress more rapidly than the first one as a consequence of end-stage liver disease.

The incidence of ALS ranges between 1.5 and 2.5 for 100,000 per year in the general population of the world (25) and the incidence of PBC is 4–40 per 100,000 people (26). Thus, the probability of co-occurrence of the two diseases in a single patient is statistically speaking very low, which may indicate common unknown mechanisms between the two diseases. The robust evidence points to a crucial role of urinary tract infection (UTI) caused by *Escherichia coli* (*E. coli*) in increasing the risk of PBC. *E. coli* infection is a key factor in the breaking of mitochondrial autoantigen immune tolerance, leading to the generation of specific AMA (27). Human PDC-E2 shares a significant homology with *E. coli* PDC-E2 which may reason for it. Besides, hepatocytes and bile epithelial cells in the liver of PBC patients express large amounts of human leucocyte antigen classes I and II molecules. Therefore, both CD4+ and CD8+ autoreactive T cells also play a crucial role in PBC (28). Thus, the pathogenesis of PBC is associated with the interaction between mitochondrial autoantigens and anti-mitochondrial antibodies and T cell-mediated toxicity (29). Changes in the immune system have also been observed in the spinal cord and cortical motor areas of ALS patients (30). The activity of CD8+ T cells could be found in both PBC and ALS. In the early stage, T cell subsets and M2 microglias are activated to prevent the neurodegenerative process. In the late stage, the activity of M1 microglia and CD8+ increases leading to decreased numbers of regulatory T cells. To some extent, the neurotoxic effect exceeds the neuroprotective effect, which results in the loss of neurons (31, 32). Association between the two diseases may be driven by dysregulation of the immune system particularly in CD8+ T cells.

The cumulative data shows that structural and functional abnormalities of mitochondria play an important role in ALS (33–35). PBC ensues from loss of mitochondrial antigen immune tolerance, and the mitochondrial autoantigens are found in all nucleated cells. Although it's said that the attack is predominantly for PDC-E2 expressed by bile epithelial cells, it is still under debate (36). Therefore, we speculate that immune attack of PBC may also impair other parts of the body, like motor neurons. Thus, (i) the normal process of electron transport chains is disturbed, causing less production of ATP (37), (ii) the destruction of Ca2+ homeostasis, resulting in synaptic dysfunction and neuronal damage (38), (iii) the apoptotic signaling is perturbed (39), leading to ALS. The hypothesis remains to be demonstrated.

With regards to treatment, UDCA is the approved medical treatment to reduce progression of disease in PBC and riluzole in ALS. However, there is no literature on specific medical doses in patients with ALS-PBC overlap syndrome. We recommended our patients to take the medications in regular doses and reexamine one time every 3 months, because most liver enzyme levels are elevated within the first 3 months of riluzole treatment (40). The patients responded satisfactorily except for intermittent nausea in the first patient. Her nausea was relieved by taking the drug in combination with food probably due to less abrupt rises in plasma concentrations. Our case report fills a gap in the researches on ALS-PBC overlap syndrome but the treatment about it has yet to be further studied. PBC is associated with immune-mediated destruction of intrahepatic bile ducts. In ALS, the immune system also plays a pivotal role. UDCA, as an immunomodulatory agent, protects cholangiocytes from bile acid toxicity in PBC patients and takes therapeutic effect on ALS (41). Recently, it has been showed that tauroursodeoxycholic acid (TUDCA) can slow progression in ALS patients (42). Compared with the first patient, our second one developed ALS after a long time with PBC, which may be related to her use of UDCA. Thus, we speculate that immune-modulating therapy for prior ALS, like UDCA, may have some protective or suppressive effect to delay the onset of motor neuron damage. However, it should be noted that this is still speculation based on a clinical phenomenon, and further studies are needed to verify the hypothesis.

## Conclusion

The coexistence of ALS and PBC indicates a relationship between the two diseases from immunological and mitochondrial aspects. The pathomechanisms of them and the effects of immune-modulating therapy at an early stage before onset of ALS symptoms remain to be elucidated combined with more clinical data.

## Ethics Statement

This study was approved by the Ethics Committee of Clinical Research of the Second Hospital of Hebei Medical University (Shijiazhuang, China). Written informed consent was obtained from the participants for the publication of this case report.

## Author Contributions

HZ: plan of the case reports, data acquisition and analysis, follow-up with patients, and writing of the initial manuscript. YL: work-up of the patients, investigation and organization of the work, manuscript review, and revision. ZL, NL, XZ, XN, TZ, and WQ: clinical investigations of patients, supplement to the argument, review, and revision.

### Conflict of Interest Statement

The authors declare that the research was conducted in the absence of any commercial or financial relationships that could be construed as a potential conflict of interest.
